# Effect of a Persian medicine formula, Glasthma, on lung function and intestinal permeability in Asthma: A triple-blind randomized controlled trial

**DOI:** 10.22038/ajp.2025.25579

**Published:** 2025

**Authors:** Ali Reza Derakhshan, Majid Mirsadraee, Amirhashem Asnaashari, Roshanak Salari, Majid Khadem-Rezaiyan, Shahin Saeidinejat, Shima Jalali, Shabnam Jalali, Shadi Gaffari

**Affiliations:** 1 *Department of Persian Medicine, School of Persian and Complementary Medicine, Mashhad University of Medical Sciences. Mashhad, Iran*; 2 *Persian Medicine Network (PMN), Universal Scientific Education and Research Network (USERN), Tehran, Iran*; 3 *Department of Internal Medicine, Faculty of Medicine, Islamic Azad University-Mashhad Branch, Mashhad, Iran*; 4 *Lung Disease Research Center, Faculty of Medicine, Mashhad University of Medical Sciences, Mashhad, Iran*; 5 *Department of pharmaceutical sciences in Persian Medicine, School of Persian and Complementary Medicine, Mashhad University of Medical Sciences. Mashhad. Iran*; 6 *Department of Community Medicine and Public Health, Faculty of Medicine, Mashhad University of Medical Sciences, Mashhad, Iran*; 7 *Department of Health Education and Health Promotion, school of Health, Mashhad University of Medical Sciences, Mashhad, Iran*; 8 *Student Research Committee, Mashhad University of Medical Sciences, Mashhad, Iran *; 9 *Department of Biology, Islamic Azad University- Damghan Branch, Damghan, Iran*

**Keywords:** Asthma, Intestinal permeability, Traditional Persian medicine Phytotherapy

## Abstract

**Objective:**

This study aimed to evaluate Glasthma, a Persian medicine herbal formulation for its efficacy and safety in managing asthma symptoms and modulating intestinal permeability.

**Materials and Methods:**

Forty randomly assigned asthma patients received Glasthma syrup (n=20) or a placebo (n=20) twice daily for 4 weeks. Respiratory symptoms, pulmonary function tests, and 5-hour urine lactulose to mannitol ratio were assessed at baseline and after 4 weeks.

**Results:**

Glasthma group exhibited significant improvements in clinical and paraclinical scores, including asthma control test (p<0.001), asthma control questionnaire 7 (p<0.007), Forced Expiratory Volume in the First Second (p<0.001), and Maximal Mid-Expiratory Flow 25-75 (p<0.002) compared to the placebo group. Lactulose and mannitol levels significantly decreased in the Glasthma group (p<0.028 and p<0.0000, respectively), with no significant changes in the ratio. No serious adverse effects were observed.

**Conclusion:**

These findings suggest that Glasthma formulation may effectively improve asthma symptoms and regulate the gut-lung axis.

## Introduction

One of the most prevalent airway inflammations and hyper-responsiveness, asthma, is accompanied by reversible bronchospasm, mucus production, and airway remodeling. The complicated interaction of genetic, epigenetic, and environmental factors frequently predisposes patients to the establishment of immune dysfunction processes (Ho, 2010).

The primary pathophysiology of the disease is airway inflammation which is caused by immune cascade activation. Increased mucus production causes thickening of the epithelium, high oxidant production exacerbates inflammation, and endothelial leak causes mucosal edema. This process eventually leads to airway obstruction (Kaveh et al., 2022; Hill and Wood, 2009;Hamzeloo-Moghadam et al., 2022; Shakrami et al., 2019). 

The specific goal is to achieve the best clinical control and reduce the risk of adverse events of asthma in the future (Boskabady et al., 2007). 

Recent reports suggest up to 80% of adults with asthma use some form of complementary medicine, commonly herbal medicine (Mirsadraee et al., 2016). The physiological effects of herbs vary depending on the multiple compounds present in a formulation (Chan et al., 2016). Based on certain researches, the anti-inflammatory and antioxidant properties of plants are effective in controlling asthmatic airway pathogenesis. The antispasmodic and bronchodilator properties of herbs may simultaneously relieve bronchoconstriction (Derakhshan et al., 2023; Janbaz et al., 2013). 

The role of the intestinal axis in the pathophysiology of respiratory diseases, especially asthma, has attracted the attention of researchers in recent years (Ananya et al., 2021). New studies emphasized the effect of intestinal permeability in the development of respiratory allergic disorders (Ho et al., 2020). 

In Persian Medicine, several formulations for asthma control and treatment have been introduced. Interestingly, some components of these formulations are known as intestinal tonic in Persian medicine's literatures. It is interesting to note that Persian medicine mentions simultaneously treating the lungs and digestive system to manage asthma symptoms, and herbal medications recommended for the treatment of asthma contain some ingredients with effects that strengthen and improve the function of the intestines. (Arzani MA, 2009; Avicenna, 2005). 

Glasthma syrup is formulated with several herbal ingredients, each of which has been individually studied for its pharmacological properties. These components include *Glycyrrhiza glabra*, *Echium amoenum*, *Cydonia oblonga*, *Ziziphus Jujuba*, and hazelnut. Previously, the effect of *Glycyrrhiza glabra* product, *Echium amoenum* and *Ziziphus jujuba* in controlling cough and improving bronchitis and asthma attacks has been confirmed (Mirsadraee et al., 2016). *Glycyrrhiza glabra* has been recognized for its anti-inflammatory, antioxidant, immunomodulatory, and anti-allergic properties (Safaeian et al., 2015). Studies have shown that *Glycyrrhiza glabra* can strengthen intestinal tight junctions and enhance the expression of genes related to intestinal barrier function (Murugan et al., 2022). Similarly, *Ziziphus iujuba* has demonstrated immunomodulatory effects and the ability to reduce inflammation and apoptosis in the gastrointestinal tract (Zhou, Guo et al. 2021). Although there is limited direct evidence of the effect of other Glasthma components on intestinal permeability, there is literature supporting the beneficial effects of quince and hazelnut in conditions associated with intestinal barrier impairment, such as chronic constipation, gastroesophageal reflux, and ulcerative colitis (Lee, Lin et al. 2012; Zohalinezhad, Imanieh et al. 2015; Lu, Yuan et al. 2022). These findings suggest that the combination of these herbal ingredients in Glasthma may contribute to its effects on improvement of intestinal permeability.

This study was designed to evaluate the efficacy and safety of Glasthma (a Persian medicine's herbal formulation) on asthma symptoms, functional lung test parameters and intestinal permeability indices. 

## Materials and Methods

### Study registry

The efficacy of Glasthma syrup on asthma was evaluated in a randomized, triple‐blind placebo‐controlled clinical study from June 2021 to March 2022. 

### Participants

Asthmatic outpatients were initially assessed and then selected based on inclusion and exclusion criteria when they visited the pulmonology department of the Ghaem educational hospital of Mashhad University of Medical Sciences, Mashhad, Iran. Inclusion criteria were being of either genders, being between 18–60 years old, being diagnosed with moderate asthma with Forced expiratory volume in the first second (FEV1) 60-80% for more than 6 months. Exclusion criteria were having cognitive diseases, history of convulsive disease, using oral or systemic corticosteroids, pregnancy or breastfeeding, hospitalization, smoking, exacerbation of the disease at the beginning of the study, hypertension, diabetes or allergy to nuts. 

### Intervention

**Figure 1:**
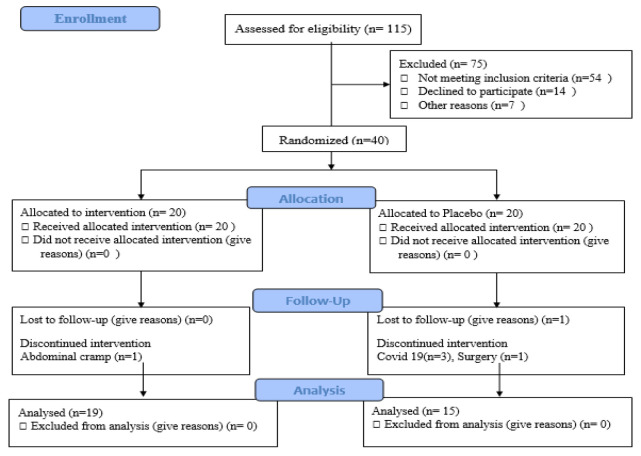
Flow diagram of study

Following the initial procedure and with the consent of the subjects, the intervention group received 15 ml of Glasthma syrup twice daily for four weeks (Figure 1). The subjects in the placebo group were given the same dosage, for the same duration, of a placebo syrup that was comparable to Glasthma syrup in terms of color, scent, and viscosity.

### Randomization and blinding

The permuted block randomization method was used to generate the random sequence. The methodologist of this project utilized the sealedenvelope.com website considering block size of 4 to provide the randomization sequence and delivered them in sequentially numbered opaque sealed envelopes for ensuring of adequate randomization concealment. Finally, after considering the inclusion and exclusion criteria, the envelopes were opened in order to assign the patients to either the Glasthma or placebo groups. The participants, investigators and statistical analyst were blinded to the assignment. 

### Herbal drug preparation

#### Collecting and extracting the plant

The Glasthma syrup was designed according to traditional Persian medical literature. All of the natural ingredients included in this formulation have been utilized based on Persian Medicine Pharmacologic books, including *Qarabadin-e-Kabir* and *Qarabadin-e-Salehi* in the treatment of asthma, in addition to the fact that these components have a positive effect on the digestive system in order to improve digestion and absorption, and improve intestinal barrier (Zarshenas et al., 2012). Then, clinical applications, dosage, safety, precautions and contraindications of each component were extracted from modern medical studies. 

Glasthma ingredients consist of Dried *Glycyrrhiza glabra* (root) (E 1355-FUMH), *Echium amoenum* (flower) (E 1352-FUMH), *Cydonia oblonga* (fruit) (E 1353-FUMH), *Ziziphus jujuba* (fruit) (E 1356-FUMH) and hazelnut (fruit) (E 1354-FUMH. All materials were verified by a certified herbalist at the herbarium of the School of Pharmacy, Mashhad University of Medical Sciences.

 The syrup was formulated and manufactured in the School of Persian and Complementary Medicine, Mashhad University of Medical Sciences. 

To prepare the Glasthma syrup, dried plants were grounded and soaked in water at 50 ^o^C for 24 hr, then filtered separately. The extracts were dried at 37 ^o^C.

The Glasthma syrup was prepared from 24.59 *Cydonia oblonga*, 11.47% Ziziphus jujuba and 11.47% *Glycyrrhiza glabra*, 19.76% *Echium amoenum* and 32.79% hazelnut in 120 ml dark colored bottles. Each 15 ml contained plants extracts (1.5 g of *Cydonia oblonga*, 0.7 g *Ziziphus jujuba* and 0.7 g *Glycyrrhiza glabra*, 1.2 g *Echium amoenum* and 2 g hazelnut) for daily consumption. Placebo syrup was made by sugar, water and certificated additives with similar color, smell and taste to Glasthma syrup.

### Standardizing the Glasthma syrup

Samples were normalized for phenolic compounds by the Folin-Ciocalteu method. The Folin-Ciocalteu method was used to standardize the sample with phenolic chemicals. Folin-Ciocalteu (100 ml) was mixed with a portion of the sample (20 ml of 10 mg/ml) and other isolated samples (20 ml of 0, 50, 100, 150, 250, and 500 mg/ml) of gallic acid. The mixture was then diluted with 300 ml of sodium carbonate (1 M), and the volume was then corrected with deionized water to 2 ml. The samples' optical absorption was evaluated by a spectrophotometer at 765 nm after two hours. The gallic acid standard curve was then plotted. Based on mg of gallic acid, the level of phenolic components in the extract was determined. The extract was then calibrated to have 143.509 mg of gallic acid per kilogram.

Investigation of the microbial culture of the Glasthma syrup, for contamination with *Escherichia coli*, *Staphylococcus aureus*, *Candida albicans*, mold and yeast counts, was done with USP32-NF27 guideline (Russo, 2010) The results showed no presence of mold, yeast, aerobic, or anaerobic bacteria.

### Measurements and outcomes

#### Procedure

Each participant answered a questionnaire at the baseline evaluation, revealing information about their age, gender, body mass index, length of illness, underlying illnesses, medical history, and drug use. At baseline and on day 28, their vital signs, lung function tests, biochemical parameters and hematology, and urinalysis were tested.

Symptoms that patients reported in the form of a survey during the study or via phone calls were used to assess side effects. Also, liver, kidney and hematologic tests were checked at baseline, and on day 28 for possible drug side effects.

### Primary outcomes

#### ACQ-7 and ACT

Clinical signs and symptoms of all subjects included in the study were assessed based on International Severe Asthma Registry (ISAR). ISAR is a globally used tool to collect anonymous, real-life information and longitudinal data of asthma patients (FitzGerald, J.M.,2020). Asthma Control Questionnaire 7 (ACQ-7) which is a part of ISAR was recorded at baseline and at the end of week 4. This questionnaire assesses the symptoms and severity of the disease over a period of one week as well as the rescue drug use and pre-bronchodilator FEV1. The score of this questionnaire varies between 0 (completely controlled) and 6 (seriously uncontrolled). Persian version of the of this questionnaire was not found, so the English version was used (Rhee et al., 2019). 

Asthma Control Test (ACT) is another component of ISAR which was completed on days 0, 28 and 56 of this study for patients. This self-report questionnaire with 5 questions examines clinical symptoms, medication use, the impact of the disease on daily life, and the patient's general opinion of asthma control in the last 4 weeks. Each question has 5 points and a score higher than 19 points shows a good control of the disease (Schatz et al., 2009). In this study, the valid and reliable Persian version was used (Juniper et al., 2005).

### Lung function evaluation

Lung function was measured by a multi-functional spirometer (Chest graph HI-801; Chest MI, Tokyo, Japan) by the volume of forced expiratory volume in the first second (FEV1) , the ratio of forced vital capacity conquered by forced expiratory volume (FEV1/FVC), maximal mid expiratory flow (MMEF25-75) (at weeks 1 and 4). 

### Intestinal permeability parameters

Particularly, urinary lactulose to mannitol ratio (L:M) is used to measure intestinal permeability. The patient received a mixture of synthetics sugar, 5 g of lactulose and 2 g of mannitol in the form of sachets. The amount of these two sugars secreted during the 5-hr urine collection serves as a proxy for the degree of intestinal permeability or malabsorption.

Determination of mannitol and lactulose in urine was done by LC-MS/MS Standard solution (6410 LC/MS/MS Triple Quad, Agilent Technologies, USA). The desired quantity of standard become dissolved and diluted in HPLC grade water with mobile phase ammonium acetate 10 mm: Acetonitrile/Water  (ACN) (80:20), column stable isotopically labeled (SIL) 25 cm, injection volume 10 µl, flow rate 0.6 ml/min(Konermann, 2017).Negative Mode result is for lactulose 341.2>>161; 341.2>>101.1 and for mannitol 181.1>>101; 181.1>>89 in Testa Quality Control Laboratory.

### Other laboratory test

Blood chemistry tests including Estimated Sediment Rate (ESR), IgE, urea, creatinine (Cr), Serum aspartate aminotransferase (AST), and serum alanine aminotransferase (ALT) were performed. complete blood count and Urine analyses. Serum biochemical and hematological parameters were measured using an automated hematological analyzer (Sysmex KX21, Sysmex Corporation, Kobe, Japan). 

### Secondary outcome

The total score of the Mini Asthma Quality of Life Questionnaire (mini-AQLQ) was calculated at the start and the conclusion of the trial to assess the participants' quality of life. This section of the ISAR instrument includes a 15-item questionnaire and is divided into 4 domains: symptoms, environment, emotions, and activities. It evaluates a 2-week period of the condition, with ratings ranging from 0 to 6 (lower is worse) (Grammatopoulou et al., 2008). 

### Statistical analysis

Data analysis was performed by independent statistical experts using IBM Statistics 16.0 software. Quantitative data is presented as median and quadratic range or mean and standard deviation. Dual data is displayed as 95% risk ratios and confidence intervals, such as symptom recurrence. A p< 0.05 was defined as statistical significance for both sequences.

Basic characteristics are described using standard statistical analysis methods. Continuous variables analyzed using t-tests, while in the case of categorical variables chi-square tests or Fisher's exact were used for analysis. The main analysis included the treatment intent, and the subject of each protocol for initial results. The latter prospective approach was used in an intention-to-treat analysis to assign missing data.

The results of quality of life scores, lung function tests, blood tests, and urine tests at the beginning of the study were compared to the results at the end of the intervention. Paired t-test was performed to compare data before and after treatment in the same group, and analysis of variance was used to compare the difference in change from baseline to end point between the two groups. Mixed-effects linear or logistic regression was used to analyze factors that influence changes in outcomes after adjusting for baseline characteristics and other variables. Any causal relationship between the intervention and side effects was considered.

According to the LM/Ratio literature (Rabbani et al., 2004), a sample size of 5 patients was obtained, with an expected loss of 20%, and 7 patients were selected. The sample size for FEV1 (Zhang et al., 2018) and ACQ7 questionnaire (Urata et al., 2002) were also the same number. Correspondingly, 7 and 6 samples were selected to take into account the 20% patient loss in each group, and 10 samples were selected, respectively. Hence, the sample size of 7 participants in each group can obtain 90% of the power of ignoring 0.05 bilateral type I error to detect a difference of 2.5 in this experiment with P-values of two sequences <0.05, which indicates significance. The sample size was increased to 20 patients in each group in order to make up for the exclusion of certain samples from the study, which was due to the inability to follow-up or manage the data and ensure quality control Forty participants were involved in our investigation (20 subjects per group).

The trial has received approval from Mashhad University of Medical Sciences (code: 991551), the Iranian National Committee for Ethics in Biomedical Research (IR.MUMS.REC.1400.076), and registration with the Iranian Clinical Trial Registry (IRCT: IRCT20210309050643N2). The subjects fulfilling eligibility criteria were recruited into the study. Before participating in the trial, each patient signed an informed consent form. Forty selected patients were randomized into two groups. The protocol of this study was published previously (Derakhshan et al., 2022).

## Results

### Procedure

Of the 115 subjects assessed for eligibility, 40 patients entered and 34 patients completed the study (Figure 1). Glasthma syrup had a 95% adherence with study medication. There were no discernible differences between Glasthma(intervention) and placebo groups in demographic characteristics, duration of disease, Pulmonary Function Test (PFT) parameters, asthma questionnaires score or laboratory parameters at baseline (Table 1). 

**Table 1:** Demographic and clinical characteristics of patients at baseline

**Variables**	**Glasthma (n = 19)**	**Placebo (n = 15)**	**p ** **value**
**Demographic details**			
Men, n (%)	10 (52.63)	6 (40.0)	0.464
Age (years)	44.84 ± 3.11	39.93 ± 3.98	0.340
BMI (kg/m^2^)	24.35 ± 0.92	24.84 ± 1.38	0.166
Job Stimulation	3(15.79)	1(6.67)	0.412
**Asthma features**			
Asthma duration (years)	13.73 ± 2.33	9.53 ± 2.65	0.243
**Lung Function parameter**			
FEV1(L)	1.94±0.83	2.30±0.62	0.172
FEV1(%)	59.76±14.48	69.21±13.26	0.057
FEV1/FVC	100.27±15.12	102.88±14.33	0.459
MEF25-75 (l/sec)	1.90±1.43	2.24125±1.20	0.111
MEF25-75(%)	69.73±31.18	80.5±32.52	0.665
FEV1(L)	1.94±0.83	2.30±0.62	0.141
FEV1(%)	59.76±14.48	69.21±13.26	0.493
**Questionnaires**			
ACQ7	18.11±6.79	19.85±6.50	0.683
ACT	11.47 ± 40.62	12.00± 1.47	0.930
AQLQ	49.70±15.61	48.78±8.61	0.661
**Laboratory test**			
Lactulose(mg/kg)	10.37±18.93	6.76±17.42	0.186
mannitol(mg/kg)	128.21±168.10	83.92±133.02	0.397
L:M	0.84±0.23	0.01±0.04	0.357
IGE(IU/mL)	161.41±182.54	221.5±188.32	0.872
Cr serum) mg/dL(	1.22±2.63	0.80±0.63	0.567
Cr urine ) mg/dL(	128.21±168.10	83.92±133.02	0.265
ESR (mm/hr)	18.90±17.25	19.73±12.34	0.229
AST (IU/L)	21.05±6.97	18.86±4.15	0.580
ALT (IU/L)	31.63±44.90	18.6±7.72	0.816
WBC(10^3^/mm^3^)	5.9±1.19	6.59±1.19	0.104
RBC (10^6^/mm^3^)	4.95±0.39	4.94±0.46	0.959
HB (g/dL)	14.26±1.70	14.54±1.11	0.598
MCV (fL)	85.56±7.19	85.94±4.49	0.859
MCH (Pgm)	28.90±3.47	29.89±2.13	0.341
Platelet(10^3^/mm^3^)	240±77.53	245.53±60.32	0.822

Data are n, n/N (%) or mean ± standard deviation (SD). BMI = body mass index. PEF= Peak expiratory ﬂow rate. FEV1: forced expiratory volume in first second; FVC: forced vital capacity; MEF25-75%: forced expiratory flow between 25% and 75%; ACQ7: Asthma control Questionnaire. ACT: Asthma control test. Mini- AQLQ: short type of Asthma Quality of Life Questionnaire. IgE: Immunoglobulin E. ESR: erythrocyte sedimentation rate. ALT: alanine transaminase. AST: aspartate aminotransferase. WBC: White Blood Cell. RBC: Red blood Cell. HB: Hemoglobin. MCV: Mean Corpuscular Volume. MCH: Mean Corpuscular Hemoglobin.

### Outcomes

#### Lung function

In the Glasthma group, the volume and percentage of FEV1 statistically improved at the end of the 4th weeks, (p=0.006 and 0.001, respectively). The same significant change was also observed in MMEF25-75 (p=0.006 and 0.002, respectively). In comparing the changes in two groups, only the FEV1 showed a statistically significant difference (p=0.041 and 0.025 respectively). No statistical difference was observed in the comparison between the Glasthma and placebo groups in terms of MMEF25-75 and FEV1/FVC (Table 2). 

### Questionnaires

The change score of the ACQ7 questionnaire in the Glasthma group had a statistically significant improvement compared to the placebo group(p=0.023).

It also improved in Asthma Control Test (ACT) questionnaire (p=0.007) and as a mid-term effect of drug, in the 8th weeks (4 weeks after ending intervention) (p=0.032) compared with placebo. The quality of life scores of patients in Glasthma group improved in comparison with patients in placebo group, but there was no significant difference between them (p=0.203) (Table 3). 

### Intestinal permeability (L:M test)

Both urine lactulose and mannitol levels were statistically decreased in Glasthma group at the end of intervention (p=0.028 and 0.000, respectively), however the decrease in L/M ratio was not significant (p=0.936). In the placebo group, the lactulose and mannitol levels and their ratio did not show a significant difference at study's end (p=0.217 and 0.674 respectively). In terms of the L/M ratio, the difference between the two groups was also not significant (p= 0.760) (Table 3). 

### Safety

Glasthma syrup was generally safe over the intervention period and we did not have withdrawal due to complications.

A total of three adverse events were reported in Glasthma group (two patients with mild abdominal upset and one with bloating), one of which was excluded from the study by personal request. Although individuals were instructed to call doctors if their asthma symptoms deteriorated, no patients required systemic glucocorticoids or hospitalizations during the trial period (Table 4).

For safety assessment of Glasthma, some serum and urine parameters were evaluated. There were no significant differences after intervention in hematological parameters (WBC, RBC, Hb, Hct, MCV, MCH, or PLT count), or biochemical tests (ALT, AST, Cr Serum and urine) (Table 3).

**Table 2:** Comparison of Peak Expiratory Flow outcomes in both groups

Variables	Glasthma (n =19)		p value*	Placebo (n = 15)		p value*	changes between week0 and 4		p value**
	Week 0	Week4		Week 0	Week4		Glasthma	placebo	
FEV1(L)	1.94±0.83	2.22±0.83	0.006	2.30±0.62	2.27±0.75	0.793	22.24 ± 5.98	4.10 ± 3.28	0.041
FEV1(%)	59.76±14.48	70.00±14.24	0.001	69.21±13.26	71.70±18.914	0.379	2.16 ± 6.05	0.03 ± 0.30	0.025
FEV1/FVC	100.27±15.12	101.43±14.93	0.713	102.88±14.33	105.28±11.96	0.346	1.16 ± 3.10	2.41 ± 2.47	0.934
MEF25-75( l/sec)	1.90±1.43	2.59±1.76	0.009	2.24125±1.20	2.77±1.44	0.105	0.69 ± 0.88	0.54 ± 0.81	0.683
MEF25-75(%)	69.73±31.18	90.4±33.76	0.002	80.5±32.52	75.86±44.39	0.802	20.67 ± 21.50	4.64 ± 5.40	0.311

*Data are presented as Mean (SD) analyzed by the Pair Sample T Test. **Data are presented as Mean (SD) analyzed by the Independent Sample Test. FEV1: forced expiratory volume in first second; FVC: forced vital capacity; MEF25-75%: forced expiratory flow between 25% and 75%

**Table 3:** Comparison of Questionnaires Parameters outcomes in both groups

Variables	Glasthma (n = 19)		p value*	Placebo (n = 15)		p value*	changes between week 0 and 4		p value**
	Week 0	Week4		Week 0	Week4		Glasthma	placebo	
ACQ7	18.11±6.79	10.44±7.28	0.000	19.85±6.50	17.35±9.11	0.076	1.16 ± 3.10	2.41 ± 2.47	0.023
ACT	11.47 ± 40.62	18.52 ± 3.73	0.000	12.00± 1.47	13.44 ± 6.93	0.352	7.05 ± 4.20	1.53 ± 6.16	0.007
AQLQ	49.70±15.61	31.29±23.21	0.004	48.78±8.61	40.78±13.26	0.104	0.95 ± 5.72	1.11 ± 2.34	0.203

*Data are presented as Mean (SD) analyzed by the Pair Sample T Test. **Data are presented as Mean (SD) analyzed by the Independent Sample Test. ACQ7: Asthma control Questionnaire. ACT: Asthma control test. Mini- AQLQ: short type of Asthma Quality of Life Questionnaire.

**Table 4:** Comparison of L:M test outcomes and other laboratory tests in both groups

Variables	Glasthma (n = 19)		p value*	Placebo (n = 15)		p value*	changes between week0 and 4		p value**
	Week 0	Week4		Week 0	Week4		Glasthma	placebo	
Lactulose(mg/kg)	10.37±18.93	0.001±0	0.028	6.76±17.42	3.14±11.46	0.334	10.38 ± 18.93	3.62 ± 14.02	0.257
mannitol(mg/kg)	128.21±168.10	28.10±28.49	0.009	83.92±133.02	61.30±88.66	0.493	100.11 ± 150.32	22.61 ± 124.27	0.110
L:M	0.84±0.23	0.052±0.22	0.686	0.01±0.04	0.01±0.03	0.335	0.03 ± 0.34	0.0078 ± 0.03	0.760
IGE(IU/mL)	161.41±182.54	272.13±450.56	0.310	221.5±188.32	269.15±258.35	0.206	101.50±81.88	47.65±35.90	0.586
Cr serum) mg/dL(	1.22±2.63	1.20±2.60	0.903	0.80±0.63	0.81±0.66	0.933	0.12	-0.08	0.073
Cr urine ) mg/dL(	128.21±168.10	28.10±28.49	0.009	83.92±133.02	61.30±88.66	0.493	0.01 ± 0.41	-0.007 ± 0.22	0.882
ESR (mm/hr)	18.90±17.25	18.36±16.34	0.867	19.73±12.34	19.66±13.23	0.950	0.54 ± 0.46	0.07 ± 0.33	0.904
AST (IU/L)	21.05±6.97	20.31±6.14	0.641	18.86±4.15	18.73±4.46	0.849	9.58 ± 13.77	0.07 ± 4.17	0.399
ALT (IU/L)	31.63±44.90	22.05±13.15	0.394	18.6±7.72	18.53±7.36	0.936	0.74 ± 47.83	0.14 ± 3.29	0.759
WBC(10^3^/mm^3^)	5.9±1.19	9.16±13.87	0.311	6.59±1.19	38.4±114.69	0.301	-3.26 ± 6.76	-3.80 ± 2.76	0.288
RBC (10^6^/mm^3^)	4.95±0.39	5.22±1.60	0.426	4.94±0.46	4.38±1.48	0.174	4.92 ± 3.66	0.56 ± 11.60	0.116
HB (g/dL)	14.26±1.70	14.37±2.42	0.739	14.54±1.11	15.03±2.46	0.292	-0/10 ± 1.45	-0.49 ± 1.50	0.485
MCV (fL)	85.56±7.19	78.13±23.08	0.155	85.94±4.49	75.18±28.05	0.159	7.42 ± 1.36	10.76 ± 1.74	0.708
MCH (Pgm)	28.90±3.47	28.46±4.29	0.617	29.89±2.13	28.07±5.72	0.198	0.44 ± 21.79	1.82 ± 27.99	0.399
Platelet(10^3^/mm^3^)	240±77.53	222.34±76.09	0.301	245.53±60.32	218.30±100.47	0.293	17.65 ± 3.80	27.23 ± 5.22	0.752

*Data are presented as Mean (SD) analyzed by the Pair Sample T Test. **Data are presented as Mean (SD) analyzed by the Independent Sample Test. L:M: lactulose to Mannitol Ratio. IgE: Immunoglobulin E. ESR: erythrocyte sedimentation rate. ALT: alanine transaminase. AST: aspartate aminotransferase. WBC: White Blood Cell. RBC: Red blood Cell. HB: Hemoglobin. MCV: Mean Corpuscular Volume. MCH: Mean Corpuscular Hemoglobin.

## Discussion

In this study, we conducted a randomized, triple-blind, placebo-controlled clinical trial to investigate the effects of Glasthma syrup, a traditional Persian medicine herbal formulation, on asthma patients' clinical symptoms, pulmonary function, and intestinal permeability. To our knowledge, this is the first investigation to explore the potential pharmacological effects of Glasthma syrup on both the respiratory and gastrointestinal systems. Our findings demonstrate several positive outcomes, which are consistent with the historical use of certain components of Glasthma in traditional Persian medicine.

In our study, FEV1 showed a significant improvement in intervention group and the FEV1 improvement was significant compared to placebo group. Asthma is described by an accentuated lower airway response to an environmental exposure, and FEV1 is a fundamental lung function parameter, for both clinical care and research. These results indicate that this preparation may have a promising effect on the underlying pathophysiology of the disease by reducing airway resistance. Besides, MMEF 25-75 showed a significant improvement in intervention group. MMEF25-75 is considered a sensitive predictive factor for small airway obstruction. This result can show the effect of Glasthma syrup on reducing airway resistance and air speediness in small airways. 

In the present study, the observed positive effects of Glasthma syrup on asthma symptoms, lung function, and intestinal permeability could potentially be attributed to the rich phytochemical composition of its ingredients. Glasthma contains a variety of phytochemicals, including phenolic compounds and tannins, which have well-documented anti-inflammatory and antioxidant properties. These compounds are known for their ability to mitigate airway inflammation, reduce oxidative stress, and modulate immune responses (Alavinezhad et al., 2018; Gholamnezhad et al., 2019; Ghorani et al., 2022).

In addition to phenols and tannins, several other phytochemicals present in Glasthma ingredients contribute to its potential therapeutic effects. Flavonoids, found in ingredients like *Echium amoenum* and *Ziziphus Jujuba*, possess anti-inflammatory properties that may alleviate bronchial inflammation (Boskabady et al., 2021). Saponins, abundant in *Glycyrrhiza glabra* are recognized for their anti-inflammatory effects (Pastorino et al., 2018). Quercetin, a flavonoid in *Cydonia oblonga* is associated with reducing airway inflammation (Aghababaei and Hadidi, 2023). Additionally, lignans in hazelnuts have antioxidant and anti-inflammatory properties (Rodríguez-García et al., 2019). These phytochemicals collectively suggest a multifaceted mechanism through which Glasthma may exert its therapeutic benefits.

Also, the association of intestinal permeability disorder with respiratory diseases, including asthma, has also been observed in previous studies (Niewiem and Grzybowska-Chlebowczyk, 2022). The most widely utilized marker for intestinal mucosal function has been the L:M ratio. This noninvasive test involves timed urine collection after particular oral dosages of mannitol and lactulose are delivered. A healthy small intestine only absorbs a tiny quantity of lactulose. But if permeability is altered, this disaccharide passes across intercellular spaces, is filtered out by the glomerulus without being reabsorbed in the renal tubules, and may subsequently be detected in the urine. Mannitol, a co-administered sugar, is absorbed by trans cellular pathways in proportion to small bowel absorptive ability (Musa et al., 2019). 

In our study, lactulose and mannitol levels in urine were significantly lower in the intervention group post Glasthma syrup consumption. This change was significantly higher than the placebo group. In other words, both lactulose and mannitol were reduced in urine, however the ratio did not change. 

In interpreting the L:M ratio finding, it is often considered that a decrease in this ratio indicates an improvement in the function of the intestinal barrier, at the same time, it should be noted that first, a decrease in the secretion of lactulose in the urine as an independent factor can indicate an improvement intestinal permeability, and second, the reduction of mannitol can also be brought on by beneficial variables such the modification of intestinal mucosa microvilli (Vojdani, 2013). In other words, lactulose is reduced due to the improvement of the function of tight junctions and mannitol due to the improvement of the intracellular function of microvilli, which leads to no significant change in the ratio of the two, while overall intestinal permeability is improved (Gan et al., 2022).

It should be noted, in a study, a decrease in the excretion of mannitol and lactulose was observed without changing their ratio. Researchers have not rejected the positive results of the intervention on intestinal permeability and have emphasized the importance of reporting sugar absorption separately (Lima et al., 2010). Among the components used in Glasthma formulation, some components such as *Glycyrrhiza glabra* and *Ziziphus jujuba* have already shown their effectiveness in improving the intestinal barrier via controlling the Caco-2 cells' tight junction protein and efflux transporter expression levels. *Glycyrrhiza glabra* strengthens intestinal tight junctions through increasing the expression of genes such as glucagon-like peptide 2 and fatty acid binding protein (Murugan et al., 2022). *Ziziphus jujuba* have shown to modulate the immune system in RAW264.7 cells via the NF-B and MAPK pathways. It attenuated gastrointestinal damage in mice by reducing inflammation and apoptosis through the inhibition of NF-B signaling (Zhou et al., 2021). 

Moreover, when considering the potential effects of these phytochemicals on intestinal permeability, they offer additional layers of complexity to the Glasthma formulation. Phenolic compounds, such as those found in *Glycyrrhiza glabra*, *Echium amoenum*, and *Cydonia oblonga*, have shown promise in fortifying the intestinal barrier (Sandoval-Ramírez et al., 2021). Tannins, another class of phytochemicals present in some Glasthma ingredients, are recognized for their astringent properties and potential to enhance gut health (Soares et al., 2020). Flavonoids and saponins in Glasthma ingredients may indirectly influence intestinal permeability by modulating immune responses and mitigating inflammatory processes within the gut (Hoskin and Coombs, 2022; Passos et al., 2022).

While these observations suggest a potential role for Glasthma ingredients in ameliorating respiratory inflammation and intestinal permeability, it is essential to note that the exact mechanisms and their impact on asthma pathogenesis remain areas of active research. To comprehensively elucidate the relationship between these phytochemicals, gut health, and asthma, future studies should explore their effects on both respiratory and gastrointestinal aspects of the disease in asthma models and patients. A deeper understanding of how Glasthma's phytochemical constituents influence both domains could hold significant therapeutic implications.

The quality-of-life questionnaire, mini AQLQ, did not show any significant change between two groups. The review of previous studies shows that there has not always been a direct relationship between the improvement of the symptoms of asthmatic patients and the improvement of the quality of life questionnaire score, so that in some studies, despite a significant improvement of the symptoms and signs of the disease, the quality of life score was not changed significantly (Yugandhar et al., 2018) . The quality-of-life scores appear to be influenced by a wide range of factors. If a question was not in the individual's lifestyle at the baseline, its score will not change at the end of the intervention, for example, people who did not engage in any extreme physical activity did not alter their results on this questionnaire. The outbreak of the Covid-19 pandemic was another confounding factor that affected the quality of life of the patients and had brought forth unanticipated limitations in the sampling process.

In summary, Glasthma syrup improved the respiratory symptoms of the patients and showed a significant effect on their lung function tests based on spirometry results. At the same time, it improved permeability in the digestive system based on L:M ratio test. The findings may be consistent with the basic theory of the connection between the gastrointestinal tract and asthma, which has been emphasized in traditional Persian medical books and has been re-considered in recent medical studies. These results should be confirmed by future larger studies. 
